# Quantifying the Availability of Vertebrate Hosts to Ticks: A Camera-Trapping Approach

**DOI:** 10.3389/fvets.2017.00115

**Published:** 2017-07-19

**Authors:** Tim R. Hofmeester, J. Marcus Rowcliffe, Patrick A. Jansen

**Affiliations:** ^1^Department of Environmental Sciences, Wageningen University, Wageningen, Netherlands; ^2^Institute of Zoology, Zoological Society of London, London, United Kingdom; ^3^Center for Tropical Forest Science, Smithsonian Tropical Research Institute, Balboa, Ancon, Panama

**Keywords:** contact rate, forest wildlife, gas theory, tick-borne disease, passage rate, host availability, *Ixodes ricinus*, remote sensing

## Abstract

The availability of vertebrate hosts is a major determinant of the occurrence of ticks and tick-borne zoonoses in natural and anthropogenic ecosystems and thus drives disease risk for wildlife, livestock, and humans. However, it remains challenging to quantify the availability of vertebrate hosts in field settings, particularly for medium-sized to large-bodied mammals. Here, we present a method that uses camera traps to quantify the availability of warm-bodied vertebrates to ticks. The approach is to deploy camera traps at questing height at a representative sample of random points across the study area, measure the average photographic capture rate for vertebrate species, and then correct these rates for the effective detection distance. The resulting “passage rate” is a standardized measure of the frequency at which vertebrates approach questing ticks, which we show is proportional to contact rate. A field test across twenty 1-ha forest plots in the Netherlands indicated that this method effectively captures differences in wildlife assemblage composition between sites. Also, the relative abundances of three life stages of the sheep tick *Ixodes ricinus* from drag sampling were correlated with passage rates of deer, which agrees with the known association with this group of host species, suggesting that passage rate effectively reflects the availability of medium- to large-sized hosts to ticks. This method will facilitate quantitative studies of the relationship between densities of questing ticks and the availability of different vertebrate species—wild as well as domesticated species—in natural and anthropogenic settings.

## Introduction

Ticks are important vectors for many pathogens that cause disease in livestock and humans (1). Many tick-borne pathogens are transmitted to ticks during a blood meal on a vertebrate host (2). The distribution of ticks over the different host species is widely assumed to be the main determinant of pathogen prevalence in ticks and hosts when (i) the tick vector parasitizes multiple host species, and (ii) these host species differ in their ability to transmit a pathogen to feeding ticks (3). Unraveling this relationship in empirical studies remains an important goal.

Gathering reliable field data on the availability of vertebrates remains challenging for species that cannot be surveyed using capture-mark-recapture techniques. Density estimation is especially difficult for medium-sized to large-bodied mammals (4), which cannot easily be live trapped, and are often shy and/or nocturnal. Many of the larger mammal species are relatively important hosts for ticks. Deer, for example, are key hosts for the ticks in the *Ixodes ricinus* complex (5). To date, field studies of ticks often rely on indirect counts or secondary sources for abundance data on these hosts. For example, one of the most extensive studies in this field used published site-level data for six out of ten species (6). Site-level density, however, may not reflect the availability of hosts to ticks at smaller spatial scales, because habitat use by mammals is usually highly heterogeneous (7).

One method to estimate the local availability of larger mammals to ticks is dung counts [e.g., Ref. (8, 9)]. While more dung does not necessarily mean more individuals, dung counts do provide a proxy for the intensity at which species frequent a given location, the factor that ultimately—more than abundance *per se*—drives availability of hosts to ticks. However, a pervasive problem with dung counts is that dung disappears at unknown and variable rates, so that the sampling period over which the dung accumulated is unknown (10). Also, dung counts cannot be used to compare availability between species, because dung deposition rates differ between host species in unknown ways. Finally, many host species do not defecate in random places as they move. For example, carnivores such as pine marten (*Martes martes*) typically deposit their scats in strategic positions to mark their territory (11). Thus, dung counts have limited value as estimates of the relative availability of vertebrate hosts to ticks.

In this paper, we introduce surveys with camera traps as an alternative method for quantifying the availability of vertebrates to ticks. Camera traps (a.k.a. trail cameras) are photo or video cameras that can be deployed in the field to photograph warm-bodied animals that pass in front. Typically, camera traps are triggered by a passive infrared sensor that detects fluctuations in thermal infrared influx. Such fluctuations arise upon the passage of animals of which the outer surface (skin or coat) is warmer (or colder) than the temperature of the background (vegetation or ground) (12). Photographic capture rates (sightings/day) derived from camera-trap images are widely used as indices of relative abundance of animals (13). Rates at which animals are captured potentially reflect the availability of hosts to ticks, as these rates are a combination of the factors underlying host availability: density, activity level, and spatial behavior of hosts (14).

Here, we describe and field test the use of camera traps to measure the relative availability of vertebrate hosts to ticks. Briefly, the approach is to deploy camera traps at random points to obtain a representative sample of photographic capture rates for all vertebrate species present, and correct these capture rates for differences in detection distance between species and habitats (15). We demonstrate that the resulting “passage rate” is directly proportional to the expected rate of contact between hosts and ticks, so can be viewed as a standardized measure of the likelihood of vertebrates passing questing ticks. To determine whether this approach was effective, we performed a field study in twenty 1-ha forest plots in the Netherlands that widely differed in fauna, and correlated relative abundance of the sheep tick *Ixodes ricinus* obtained from drag sampling with passage rates of hosts as estimated with camera trapping. We used principal component analysis (PCA) to assess whether (i) host passage rates reflected known differences in wildlife assemblage composition between sites, and whether (ii) host passage rates were correlated with the relative abundances of three life stages of the sheep tick in a manner consistent with expectations based on known associations with host species. Specifically, we expected tick densities to increase with the availability of deer, which are considered key hosts (8, 16).

## Material and Methods

### Development of the Method

Most tick species that are of medical or veterinary importance wait for a passing animal in the vegetation, like sit-and-wait predators, in a position known as “questing” (2). While questing, ticks cling to leaves by their third and fourth pair of legs, and hold the first pair of legs outstretched, waiting to grasp and climb on to any passing host (17). Ticks detect hosts by CO_2_, body odors, body heat, moisture, and vibrations (18). Thus, successful attachment to a host depends on the frequency and proximity of passage, similar to a predator in ambush (19). Only host animals that walk on the ground are available to ticks (2).

Camera traps are similar to ticks (and other sit-and-wait predators) in that they detect and capture passing animals (12). Animal detection by cameras is, like animal detection by ticks, a function of the frequency and proximity of passage. The approach of using camera traps to assess host availability to ticks is based on the idea that cameras positioned at the questing height of ticks quantify the frequency and proximity of animal passage, which together determine the rate at which ticks encounter hosts. We assume that the likelihood of a questing tick to encounter a certain host species is proportional to the rate at which hosts pass random points at questing height.

Using gas-model theory, we first show that animal passage rate from cameras is theoretically proportional to the rate of contact between animals and ticks. The contact rate between camera traps and animals (*R*_cam_) can be calculated as:

(1) $$R_{\text{cam}}=\frac{D\cdot v\cdot r_{\text{cam}}\cdot \left(2+\theta_{\text{cam}}\right)}{\pi }$$

where *D* is the density at which the animal occurs, *v* the average day range of the animal, *r*_cam_ the average distance from the camera at which animals are detected (detection distance), and θ_cam_ the angle of the circular sector within which animals are detected (detection arc) (20). In contrast to camera traps, ticks can detect animals from all directions. If we assume that animals walk randomly in relation to the position of ticks, the contact rate between ticks and animals (*R*_tick_) is best described by a gas model where both “sensors” and “signals” are circular (21):

(2) $$R_{\text{tick}}=D\cdot v\cdot 2\cdot r_{\text{tick}}$$

As ticks and cameras are positioned in the same system, *D* ·*v* is constant, so we can express *R*_tick_ as a function of *R*_cam_:

(3) $$R_{\text{tick}}=\frac{R_{\text{cam}}\cdot 2\cdot r_{\text{tick}}\cdot \pi }{r_{\text{cam}}\cdot \left(2+\theta_{\text{cam}}\right)}$$

The accuracy of *R*_tick_ critically depends on the careful positioning of the camera traps—horizontal at questing height—and the accurate estimation of *r*_cam_ and θ_cam_. Multiple methods are available for estimating these parameters (15, 22, 23). The simplest method is to define a line of sight in the view of the camera trap, measure at what distance animals cross that line, and then fit a function to these distances for each species to estimate *r*_cam_, as explained in Hofmeester et al. (15). Counting only the passages over that line effectively reduces θ_cam_ to zero, simplifying the equation:

(4) $$R_{\text{tick}}=\frac{R_{\text{cam}}\cdot r_{\text{tick}}\cdot \pi }{r_{\text{cam}}}$$

As *r*_tick_ is a constant, *R*_tick_ is directly proportional to $\frac{R_{\text{cam}}}{r_{\text{cam}}}$, the “passage rate” adjusted for variation in detection distance. By estimating *r*_cam_ per species and habitat type (15) passage rates are standardized and thus comparable between species and sites. This means that passage rates of different species can be summed to estimate the availability of groups of species, such as specific functional or taxonomic groups.

The average passage rate of vertebrates in a study plot can be estimated by placing cameras at random points, such that a representative sample of the study area is obtained. In practice, the camera is mounted on a tree at or near a computer-generated random point, or on a pole that is driven into the soil. The average passage rate can be correlated with estimates of tick density or infection prevalence in ticks, as obtained by sampling ticks in the same plot, either during the same period or after a time lag when the focal tick species has a multi-year life cycle (2).

### Field Test

We tested this method by sampling 20 1-ha plots in 19 forests across the Netherlands with contrasting faunas, calculated average passage rate, and correlated these with abundance estimates of the sheep tick (*Ixodes ricinus*), a three-host species that quests in the vegetation (5). Larvae of the sheep tick feed mainly on small mammals, nymphs feed on medium-sized birds and mammals of any size, and adults feed mainly on deer (24). While the density of sheep ticks is dependent on the availability of many different host species, several studies have shown that tick density is especially correlated with deer density (8, 16).

All sampling plots were located in forests that had either pedunculate oak (*Quercus robur*), Scots pine (*Pinus sylvestris*), or a combination of these (mixed forest) as dominant tree species (Table 1). Forests were selected based on published distribution maps of mammals in the Netherlands (25). All plots were >5 km apart except for two plots at Enkhout, which were just 150 m apart; one of these was located in a 3-ha stand fenced three years prior to the study to create a situation without large mammals. We sampled eleven plots for vertebrates and ticks in 2013 and nine plots in 2014.

**Table 1:** Characteristics of the research sites and sampling effort (camera days).

Site	Vegetation		Coordinates^b^		Year	Effort (days)
	Overstory	Undergrowth^a^	Latitude	Longitude		
Amsterdamse Waterleiding Duinen	Mixed forest	*Calamagrostis epigejos*	52°20′36″N	4°33′58″E	2014	492
Bergherbos	Mixed forest	*Deschampsia flexuosa*	51°55′14″N	6°14′30″E	2013	504
Buunderkamp	Scots pine forest	*Vaccinium myrtillus*	52°00′56″N	5°44′50″E	2013	504
Duin en Kruidberg	Mixed forest	*Calamagrostis epigejos*	52°26′16″N	4°36′18″E	2013	504
Deelerwoud	Scots pine forest	*Vaccinium myrtillus*	52°05′51″N	5°56′42″E	2014	504
Enkhout	Scots pine forest	*Vaccinium myrtillus*	52°16′25″N	5°54′49″E	2013	495/504^c^
Herperduin	Mixed forest	*Molinia caerulea*	51°45′33″N	5°36′53″E	2014	504
Halfmijl	Mixed forest	*Molinia caerulea*	51°25′23″N	5°19′09″E	2013	504
Kremboong	Pedunculate oak forest	*Dryopteris dilatata*	52°45′13″N	6°31′16″E	2013	504
Maashorst	Mixed forest	*Deschampsia flexuosa*	51°42′44″N	5°35′24″E	2014	504
Pettemerduin	Pedunculate oak forest	*Polypodium vulgare*	52°46′33″N	4°40′19″E	2014	499
Planken Wambuis	Scots pine forest	*Vaccinium myrtillus*	52°01′54″N	5°48′36″E	2013	441
Rheebruggen	Pedunculate oak forest	*Dryopteris dilatata*	52°46′60″N	6°17′44″E	2014	504
Schoorlse Duinen	Mixed forest	*Molinia caerulea*	52°41′47″N	4°40′01″E	2013	504
Stameren	Mixed forest	*Deschampsia flexuosa*	52°03′38″N	5°21′01″E	2014	486
Valenberg	Scots pine forest	*Vaccinium myrtillus*	52°15′33″N	5°48′47″E	2014	391
Vijverhof	Mixed forest	*Deschampsia flexuosa*	52°09′43″N	5°13′43″E	2013	507
Vledderhof	Pedunculate oak forest	*Dryopteris dilatata*	52°52′46″N	6°14′25″E	2014	504
Zwanemeerbos	Pedunculate oak forest	*Pteridium aquilinum*	53°00′46″N	6°45′19″E	2013	504

*^a^The most dominant plant species in the herbaceous layer*.

*^b^Coordinates as measured with a handheld GPS (Garmin eTrex 20) in the plot center*.

*^c^The number of camera trapping days outside of the exclosure (left) and inside the exclosure (right)*.

As baseline for comparison, we determined the expected presence of hosts based on habitat preference and distribution of the different medium-sized to large mammals in the Netherlands [Table 2; Ref. (25)]. We only included species known to be parasitized by the sheep tick (26) and excluded small mammals—bank vole (*Myodes glareolus*), weasel (*Mustela nivalis*), and wood mouse (*Apodemus sylvaticus*)—as species of this size are difficult to capture using camera traps (23), and because, based on their distribution patterns, these species occurred in all 20 plots.

**Table 2:** Presence of mammal species in the 20 study plots as expected based on habitat preference and published distribution maps (left) and as detected by camera traps in this study (right).

Group^a^	Plot	Carnivores					Deer			Lagomorphs		Others		
		Badger (*Meles meles*)	Pine marten (*Martes martes*)	Polecat (*Mustela putorius*)	Red fox (*Vulpes vulpes*)	Stone marten (*Martes foina*)	Fallow deer (*Dama dama*)	Red deer (*Cervus elaphus*)	Roe deer (*Capreolus capreolus*)	Hare (*Lepus europaeus*)	Rabbit (*Oryctolagus cuniculus*)	Hedgehog (*Erinaceus europaeus*)	Red squirrel (*Sciurus vulgaris*)	Wild boar (*Sus scrofa*)
1	Enkhout (exclosure)	0/1	1/1										1/0	
	Pettemerduin		1/0	1/0	1/1					1/1	1/0	1/1		
	Schoorlse Duinen		1/1		1/1					1/1	1/0	1/0	1/0	

2	Buunderkamp	1/1	1/1		1/1				1/1				1/1	0/1
	Stameren	1/1	1/1		1/1				1/1				1/1	
	Vijverhof	1/1	1/0		1/1				1/1	1/1			1/1	

3	Bergherbos	1/1	1/1	1/0	1/1	1/1			1/1	1/1	1/0	1/0	1/1	
	Herperduin	1/1		1/1	1/1				1/1	1/1	1/1	1/0	1/1	
	Halfmijl			1/1	1/0	1/1			1/1	1/1	1/0	1/1	1/0	
	Kremboong	1/1	1/0	1/0	1/1	1/0			1/1	1/1	1/0	1/0	1/0	
	Maashorst	1/1		1/0	1/1	1/1			1/1	1/1	1/1	1/0	1/1	
	Rheebruggen	1/1	1/1	1/1	1/1	1/1			1/1	1/1	1/0	1/0	1/0	
	Vledderhof	1/1	1/1	1/1	1/1	1/1	0/1		1/1	1/1	1/0	1/0	1/0	
	Zwanemeerbos			1/0	1/1	1/1			1/1	1/1	1/0	1/1	1/1	

4	Amsterdamse Waterleiding Duinen		1/1	1/1	1/1		1/1		1/0	1/0	1/0	1/1	1/1	
	Duin en Kruidberg		1/1	1/0	1/1		1/1		1/1	1/1	1/0	1/1	1/0	

5	Deelerwoud	1/1	1/1		1/1		1/1	1/1	1/0				1/0	1/1
	Enkhout	1/1	1/1		1/1		1/1	1/1	1/1				1/0	1/1
	Planken Wambuis	1/1	1/0		1/1			1/1	1/1	1/1			1/0	1/1
	Valenberg	1/1	1/1		1/1		1/1	1/1	1/1				1/0	1/1

*^a^Plots are grouped based on their expected vertebrate assemblage. Plots without any ungulates (Group 1); plots with roe deer presence and a relatively species poor assemblage (expected species richness <7; Group 2); plots with roe deer presence and a relatively species rich assemblage (expected species richness >7; Group 3); plots with roe deer and fallow deer presence expected (Group 4); and plots with roe deer, red deer and wild boar presence expected (Group 5)*.

### Camera Trapping of Mammals

We measured the availability of hosts throughout March–November, the main activity season of the sheep tick in the Netherlands (27), by running camera traps at 18 random points per plot (as rule of thumb, in homogenous areas, the average passage rate stabilizes as the number of sample points approaches 20) (28). Two camera traps (HC500, Reconyx Inc., Holmen, WI, USA.) were deployed at random points in each plot during nine consecutive rounds of four weeks. Points sampled simultaneously were >30 m apart, as to reduce the likelihood of any animal walking past both cameras in a short amount of time. Thus, we sampled 18 points per plot for a total of 504 sampling days (Table 1). Cameras were placed on a tree nearest to a computer-generated random point, at 40 cm above the ground with the view parallel to the ground, without bait or lure. We set the camera traps to the highest sensitivity, to take a series of ten pictures when triggered with no delay, and to take time-lapse photos every 12 h to generate a record of camera functioning. We used a semi-automated image-processing tool (28) in which sequences were combined per event.

We quantified *r*_cam_ for each species and habitat type using the line-transect method (15) to control for variation in detectability between species and sites. This method involved the placement of a line of markers in the center of the view at distance intervals of 2.5 m. For each animal passage, we recorded the species and distance interval at which the animal crossed the line. Using these frequency data, we estimated *r*_cam_ for all mammal species per habitat type (Table 3) using a point model with a detection probability, described by a half-normal function, and with log10-transformed body mass as covariate (15). Estimates of *r*_cam_ were used to estimate passage rate per species per camera. For each plot, we then calculated the average passage rate (m^−1^⋅day^−1^) per species by using the arithmetic mean over sampling points. Furthermore, we combined passage rates of related species into passage rates of taxonomic groups by summing the detection-corrected species- and site-specific passage rates.

**Table 3:** Estimated effective detection distances (*r*_cam_) of species by camera traps in this study, estimated using a point model with a half-normal detection probability fitted to recorded passage distances, with log_10_-transformed body mass as covariate, and by habitat type.

Species	Body mass^a^ (kg)	*r*_cam_ (m) by Habitat^b^				
		CE	DF	FE	MC	VM
**Carnivores**						
Badger (*Meles meles*)	11.8		5.4	5.4	5.5	4.5
Pine marten (*Martes martes*)	1.3	4.5	3.7	3.5	3.4	3.0
Polecat (*Mustela putorius*)	1.0	4.4		3.4	3.2	
Red fox (*Vulpes vulpes*)	4.8	5.0	4.6	4.5	4.5	3.8
Stone marten (*Martes foina*)	1.7		3.9	3.7	3.6	
**Deer**						
Fallow deer (*Dama dama*)	57.2	6.2		7.2		5.9
Red deer (*Cervus elaphus*)	240.9					7.6
Roe deer (*Capreolus capreolus*)	22.5	5.7	6.0	6.1	6.3	5.0
**Lagomorphs**						
Hare (*Lepus europaeus*)	3.8	4.9	4.5	4.3	4.3	3.7
Rabbit (*Oryctolagus cuniculus*)	1.6		3.8		3.5	
**Rest**						
Hedgehog (*Erinaceus europaeus*)	0.8	4.3		3.2	3.0	
Red squirrel (*Sciurus vulgaris*)	0.3	4.0	3.0	2.7	2.5	2.4
Wild boar (*Sus scrofa*)	84.5					6.3

*CE, Calamagrostis epigejos; DF, Deschampsia flexuosa; FE, fern species (Dryopteris dilatata, Polypodium vulgare, or Pteridium aquilinum); MC, Molinia caerulea; and VM, Vaccinium myrtillus*.

*^a^Body mass values were taken from the PanTHERIA database (29)*.

*^b^Dominant herbaceous species in the 1-ha forest plot*.

### Drag Sampling of Ticks

We estimated the abundance of questing ticks by blanket dragging six times in each plot, once every four weeks during April–September, the main activity season for the sheep tick in the Netherlands (30). During each session, a cotton cloth of 1 m^2^ was dragged for twenty transects of 10 m, resulting in 200 m^2^ per session as in (31). We dragged for ticks only during conditions with dry weather with a temperature above 10°C and dry vegetation, following the recommendations of Mejlon and Jaenson (32) and Randolph (2). After each transect, we counted all larvae, nymphs, and adult sheep ticks on the bottom side of the cloth and used these numbers to calculate the average tick density per 100 m^2^ for each life stage.

### Analyses

We used principal component analysis (PCA) to quantify differences in passage rates of wildlife across plots, with the *vegan* package in R 3.2.3 (33, 34), as this multivariate analysis allowed us to examine relationships in host passage rate of the whole mammal community in a single model (35). We did this both for individual species and for taxonomic groups. To determine whether camera trapping accurately captured the wildlife communities of the sampling plots, we calculated the percentage overlap between the expected and observed presence of species (Table 2).

Second, we used PCA to quantify differences in tick densities across the plots by life stage, and related the outcome to the passage rates of vertebrate hosts. Here, the multivariate approach allowed us to model the three life stages of the sheep tick simultaneously to explore general patterns for the species, which may be more informative than specific correlations per life stage. We tested for correlations between the availability of specific vertebrate species and the sheep tick composition of a plot using 999 permutations in the *envfit* function of the *vegan* package (33, 36). Specifically, we verified whether variation in density of different life stages of ticks between sites was explained by passage rates of deer, which are known as key hosts (8, 16).

## Results

Camera traps detected a total of thirteen medium-sized to large mammal species in the 20 forest plots (Table 3). Overall, the species presence as determined with camera trapping was similar (72%) to what was expected based on habitat preference and distribution patterns (Table 2). We recorded fallow deer and wild boar in two plots that were located outside the published distribution patterns, and we recorded a badger inside the exclosure at Enkhout, which had dug its ways underneath the fence. Only the four smallest mammal species—polecat, rabbit, hedgehog and red Squirrel—were recorded in fewer plots than expected (37% similarity), while the three largest species—fallow deer, red deer, and wild boar—were recorded in all plots where they were expected (100% similarity; Table 2). Detection distance, *r*_cam_, differed considerably between species and vegetation types (Table 3).

**Figure 1:**
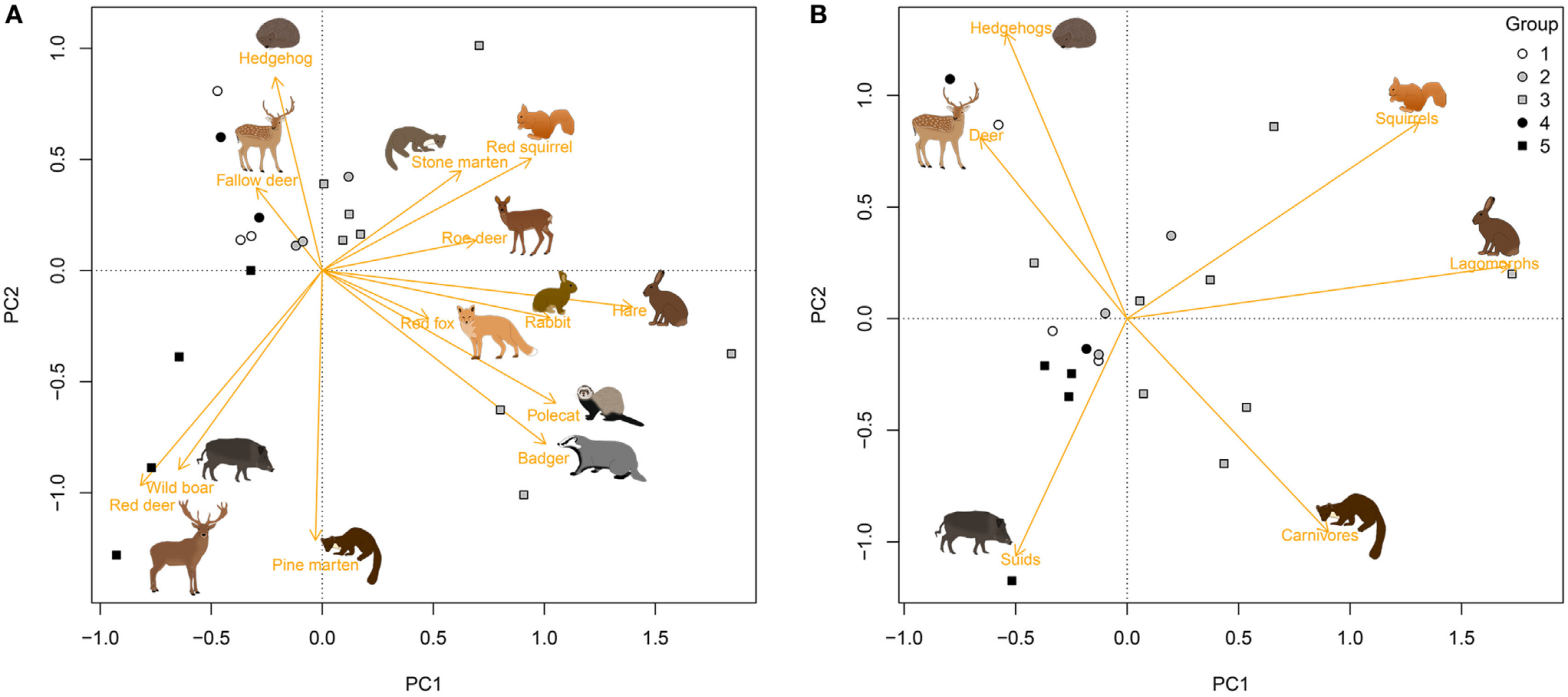
Differences in passage rate of medium- to large-sized mammals among 20 forest plots in the Netherlands, estimated with camera traps placed at 18 random points per plot. Graphs show the first two axes of a Principal Component Analysis (PCA) for **(A)** species and **(B)** taxonomic groups (orange vectors). White symbols refer to plots without ungulates, gray symbols to plots in which roe deer (*Capreolus capreolus*) was the only ungulate species, and black symbols to plots with two to four ungulate species. See Table 2 for an explanation of group numbers.

Principal Component Analysis of passage rates reflected the large variation between plots in the composition of the host assemblage (Figure 1A), where the first three axes of the PCA explained 57% of the variation (Table S1 in Supplementary Material). Plots grouped together as expected, where the first PCA axis separated the plots in groups 1, 4, and 5 from those in groups 2 and 3, and the second PCA axis separated groups 4 and 5 (Figure 1A). Much of the variation in passage rate was retained after combining capture rates of host species by taxonomic group (Figure 1B), where the first two axes of the PCA explained 56% of variation between forest plots.

**Figure 2:**
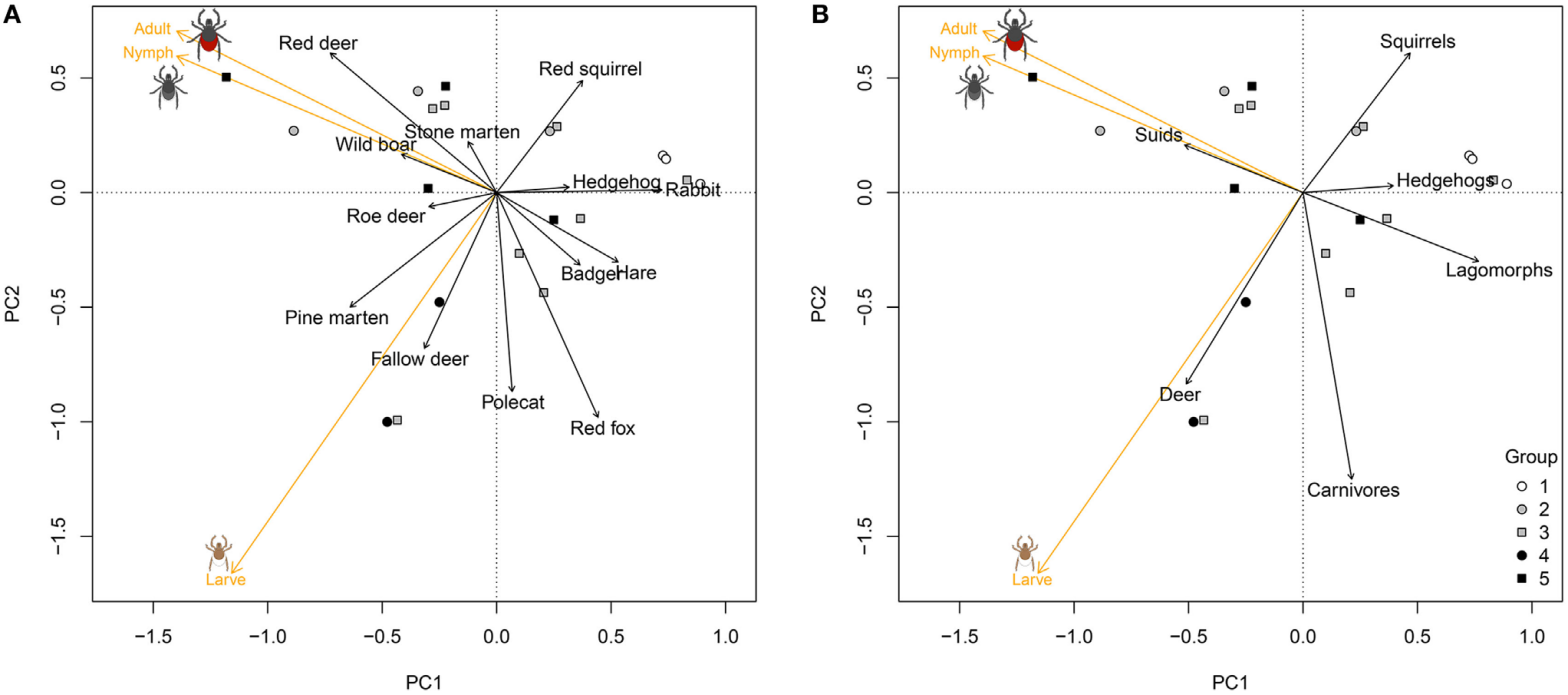
Variation in estimated density of three life stages of the sheep tick (*Ixodes ricinus*) across 20 forest plots in the Netherlands, estimated with drag sampling, in relation to the passage rates of medium- to large-sized mammals in the same plots. Graphs show the first two axes of a principal component analysis (PCA) of differences in tick density across plots by life stage (orange vectors), correlated with the passage rates of host species **(A)**, and taxonomic groups of vertebrates **(B)** (black vectors). Symbols as in Figure 1.

The density of questing sheep ticks also varied widely across the 20 forest plots. The variation was well described by the first two axes of the PCA (87% of variation explained), where the first axis captured the variation between plots with very low tick densities for all three stages and plots with high tick densities for all three stages. The second axis separated plots with high densities of larvae from plots with high densities of nymphs and adults (Figure 2). Plots without deer (Group 1) had the lowest tick densities, plots with high passage rates of ungulates the highest (Figure 2). At the level of host species, differences in tick density between forest plots were best explained by the passage rates of red fox (*p* = 0.06, *R*^2^ = 0.28) and red deer (*p* = 0.12, *R*^2^ = 0.22) (Figure 2A). At the level of taxonomic groups of hosts, differences in tick density were best explained by passage rates of carnivores (*p* = 0.08, *R*^2^ = 0.25), and deer (*p* = 0.21, *R*^2^ = 0.15): tick density increased with passage rates of deer and decreased with passage rates of carnivores (Figure 2B).

## Discussion

Quantifying the availability of vertebrate hosts to ticks is important for our understanding of the dynamics of ticks and tick-borne diseases but has been challenging in field settings. Here, we have described a procedure in which host availability to ticks is estimated as the rate at which hosts pass randomly placed camera traps. In a field test of this procedure across 20 forest plots in the Netherlands, passage rates largely reflected the mammal assemblage that was expected based on habitat preference and published distribution maps of the species. Moreover, densities of the sheep tick (*Ixodes ricinus*) were correlated with passage rates of hosts in a manner expected based on literature. Therefore, we conclude that measuring host passage rates with camera traps is an effective method to quantify the relative availability of hosts to ticks.

Camera trapping recorded all thirteen species of mammal that we expected to find (Table 2). Large species such as red deer (*Cervus elaphus*) and wild boar (*Sus scrofa*), which have a restricted range in the Netherlands (25), were recorded in all sites where they were expected to occur. However, smaller species such as red squirrel (*Sciurus vulgaris*), hedgehog (*Erinaceus europaeus*), rabbit (*Oryctolagus cuniculus*), and polecat (*Mustela putorius*) were often missed in plots where they were expected based on habitat preference and distribution. This may be partly due to the relatively small size of these species, giving lower detection distance (Table 3), and so lower overall probability of detection. However, there are likely additional, species-specific reasons for this result. Red squirrels only occasionally visit the ground (37), which means that they are rarely available for detection by camera traps. However, this also means that they effectively have low availability as hosts to ticks, which is precisely what the passage rates reflect. The other three species prefer half-open landscapes and enter forest only occasionally (25), which might explain the low passage rates or non-detection in multiple plots. In other words, passage rate appeared to accurately reflect the effective local availability of mammalian hosts to ticks.

Sheep tick density correlated with host passage rate in a manner that was consistent with expectations (Figure 2). Sheep tick density was higher in areas with higher availability of deer—red deer, roe deer (*Capreolus capreolus*), and fallow deer (*Dama dama*)—which agrees with previous studies that found a positive correlation between the number of ticks and densities of roe deer and red deer (8, 16). Our finding that sheep tick density was lower in areas with a high availability of red fox (*Vulpes vulpes*) was consistent with a study that found a negative correlation between densities of the black-legged tick (*Ixodes scapularis*) and foxes in north–western USA (38).

The presented method considers variation in host availability to ticks in two dimensions. However, the availability of hosts to ticks will also vary in a third dimension: the height relative to the ground surface. The three life-stages of the sheep tick quest at different heights: 0–29 cm for larvae, 30–59 cm for nymphs, and 60–79 cm for adults (32), which is also dependent on vegetation height. Camera traps mounted at 40-cm height most likely capture all animals passing from ground level up to approximately 2 m high. Only small animals (<40 cm high) that pass right before the camera might be missed, but this can be incorporated in the detection probability function for estimating the detection distance *r*_cam_ (23). Therefore, camera traps likely capture all animals that are available, but not all animals passing will be available for all life stages of the ticks. Performing analyses per tick life stage, including only species that are available at the height at which the life stage quests might resolve this issue, but remains to be tested.

More in general, camera traps work well to measure passage rates of medium-sized and large mammals, but miss small mammals and birds when mounted at knee level. This is unfortunate, because small mammals are key hosts for the larval stage of the sheep tick (24). Camera traps in our field test rarely captured small mammals, although live trapping showed that rodents were present in all but one plot (39). This is likely due to their small size, which makes it difficult for the passive infrared sensor of the camera traps to detect them (15). Alternative camera set-ups to study small mammals exist, either using the camera in combination with a conventional live trap (40) or using the camera in a specifically constructed box (41), but these yield estimates that cannot simply be combined with passage rates of larger hosts. A possible solution is to simply mount the camera traps closer to the ground, which increases the probability of detecting small mammals and birds (23, 42). This would allow measuring passage frequencies of all hosts with a single method. One drawback, however, is that placement closer to the soil surface is at the cost of depth in the view, which will make the estimation of *r*_cam_ and θ_cam_ for larger species harder.

The method could be further improved by considering movement patterns of hosts. For now, we have weighted every animal passage equally. However, the opportunity for a tick to contact a host will differ between animals running past in a matter of seconds, and animals foraging in front of the camera, moving back and forth for several minutes. This difference could be taken into account by measuring the actual movement distance and/or time spent in front of the camera, as was done by Rowcliffe et al. (43). Such refinement should further increase the value of the passage rate as an estimate of host availability to ticks.

Our example setup did not allow us to determine fluctuations in host availability over the seasons, because the number of camera traps present in each site at any one moment was too low while the deployment time of each camera trap was rather long. Thus, we could only analyze yearly averages, and missed fluctuations in host availability or tick density throughout the season. This could be improved by increasing the number of camera traps per site and by shortening the deployment time for each individual camera trap, to for example 7 days rather than 28 days, in order to increase the number of deployments in a site. In that way, bias due to camera placement within each season is reduced and analyses can be performed on a temporal scale.

Our index of passage rate assumes that ticks are randomly distributed compared to the movement of their hosts. As ticks quest close to the spot where they dropped off the host during their previous blood meal or the blood meal of their mother, this assumption might not be valid in relation to the species of host of that previous blood meal. However, for very generalist species such as the sheep tick, the distribution of ticks in relation to the movement of all other host species might be random. More studies relating the distribution of questing ticks to the movement of hosts are needed to further test this assumption.

Furthermore, while we have demonstrated that passage rates give sensible results as an index of the strength of interaction between ticks and their hosts, the approach also has potential as a quantitative estimator of contact rate, a key parameter needed in models of tick population dynamics (44, 45). This could be done by using equations 3 or 4 with an estimate of *r*_tick_ (distance of approach between host and tick required for a tick to attach to the host) to predict host-tick contact rate. This potentially offers a new way of parameterizing disease transmission models. However, for this approach to be credible, further work is required to derive robust estimates of host-tick detection distance.

In conclusion, the availability of medium- to large-sized mammalian hosts to ticks can be estimated using randomly-placed camera traps. We show that these passage rates are directly proportional to the encounter rate between hosts and ticks, which can be used to model tick population dynamics in situations with differing host availability. Passage rates estimated with camera traps also have potential as estimates of host availability to other parasites or pathogens that are encountered in the environment, such as *Francisella tularensis* or Puumala hanta virus (46, 47). Furthermore, the camera-trap data that must be acquired to estimate passage rates can also be used to estimate other parameters relevant to disease transmission such as the probability of different host species meeting in space or time (48).

## Author Contributions

TH and PJ jointly designed the study, performed the analyses, and wrote the manuscript; TH collected the data; JR provided the theoretical underpinning of the method; all authors approved the final version of the manuscript.

## Conflict of Interest Statement

The authors declare that the research was conducted in the absence of any commercial or financial relationships that could be construed as a potential conflict of interest.
