# Supplementary material for: Quantifying the Availability of Vertebrate Hosts to Ticks: A Camera-Trapping Approach
Source: Front Vet Sci. 2017 Jul 19;4:115. doi: 10.3389/fvets.2017.00115 (PMC5515830; doi:10.3389/fvets.2017.00115)
Supplement: Supplementary file 1 [file Table_1.DOCX]

Table S1. Principal Component Analysis (PCA) scores for the availability of hosts to ticks in the twenty forest plots.

|  | PC1 | PC2 | PC3 |
| --- | --- | --- | --- |
| Eigenvalue | 3.51 | 2.39 | 1.57 |
| Cumulative proportion explained | 0.27 | 0.45 | 0.57 |
| *Species* |  |  |  |
| Badger (*Meles meles*) | 0.72 | -0.56 | 0.00 |
| Fallow deer (*Dama dama*) | -0.21 | 0.27 | 0.57 |
| Hare (*Lepus europaeus*) | 1.00 | -0.12 | 0.09 |
| Hedgehog (*Erinaceus europaeus*) | -0.15 | 0.62 | 0.28 |
| Pine marten (*Martes martes*) | -0.02 | -0.87 | -0.21 |
| Polecat (*Mustela putorius*) | 0.75 | -0.43 | 0.14 |
| Rabbit (*Oryctolagus cuniculus*) | 0.73 | -0.15 | 0.64 |
| Red deer (*Cervus elaphus*) | -0.58 | -0.69 | 0.02 |
| Red fox (*Vulpes vulpes*) | 0.34 | -0.15 | 0.35 |
| Red squirrel (*Sciurus vulgaris*) | 0.67 | 0.36 | 0.06 |
| Roe deer (*Capreolus capreolus*) | 0.49 | 0.10 | -0.76 |
| Stone marten (*Martes foina*) | 0.45 | 0.32 | -0.55 |
| Wild boar (*Sus scrofa*) | -0.46 | -0.64 | 0.06 |
